# Development of next generation nanomedicine-based approaches for the treatment of cancer: we've barely scratched the surface

**DOI:** 10.1042/BST20210343

**Published:** 2021-10-28

**Authors:** Shannon R. Tracey, Peter Smyth, Caroline J. Barelle, Christopher J. Scott

**Affiliations:** 1 The Patrick G Johnston Centre for Cancer Research, Queen's University, 97 Lisburn Road, Belfast BT9 7AE, U.K.; 2 Elasmogen Ltd, Liberty Building, Foresterhill Health Campus, Aberdeen AB25 2ZP, U.K.

**Keywords:** cancer, conjugation, drug delivery, nanomedicine, surface functionalisation, targeted therapeutics

## Abstract

Interest in nanomedicines has grown rapidly over the past two decades, owing to the promising therapeutic applications they may provide, particularly for the treatment of cancer. Personalised medicine and ‘smart’ actively targeted nanoparticles represent an opportunity to deliver therapies directly to cancer cells and provide sustained drug release, in turn providing overall lower off-target toxicity and increased therapeutic efficacy. However, the successful translation of nanomedicines from encouraging pre-clinical findings to the clinic has, to date, proven arduous. In this review, we will discuss the use of nanomedicines for the treatment of cancer, with a specific focus on the use of polymeric and lipid nanoparticle delivery systems. In particular, we examine approaches exploring the surface functionalisation of nanomedicines to elicit active targeting and therapeutic effects as well as challenges and future directions for nanoparticles in cancer treatment.

## Introduction

Cancer is one of the most significant global health burdens and the leading cause of mortality worldwide. Despite notable improvements in cancer treatment, one of the most prominent challenges affecting the development of novel therapeutics is controlling the biological fate of the active pharmaceutical ingredient (API) following administration. Current first-line therapies, specifically within the field of cancer, are often limited by poor stability, solubility and rapid metabolism, resulting in poor pharmacokinetics [1]. Furthermore, major off-target effects result in adverse toxicities in healthy cells, in turn contributing to reduced treatment tolerability and poorer patient outcomes. Failure of treatment is therefore frequently attributable to inadequate drug delivery to the tumour site, as opposed to the efficacy of the drug itself.

One means of overcoming this issue is through incorporation of the drug within nano-based drug delivery systems. Nanomedicine is rooted in the concept of developing improved formulation of difficult/insoluble APIs, but is now an exponentially evolving interdisciplinary field. A substantial amount of research has been carried out investigating nanoparticle design and development, including assessment of passive targeting/accumulation, active targeting, and triggered drug release.

A particular benefit of nanoparticles is the opportunity to entrap APIs within their structure, providing a transient barrier for labile therapeutics as well as enhancing the bioavailability of drugs which ordinarily exhibit poor solubility. Thus nanocarriers can be used to protect drug cargos while in circulation, improving both pharmacokinetic and pharmacodynamic profiles of APIs [2–4]. Furthermore, nanoparticles have the ability to preferentially localise within the tumour tissue due to the increased permeability of the tumour microenvironment (TME) neovasculature, resulting in sustained drug release [5–7]. Essentially this means that the therapeutic window of the payload agent can be broadened by nanoformulation through simultaneous reduction in systemic toxicity and enhancement of drug efficacy. These properties are particularly appealing within the field of oncology for sustained delivery of potent cytotoxic chemotherapeutic agents [8–15].

Although the majority of research has focused upon the entrapment of cytotoxic agents, nanoparticles for the intracellular delivery of proteins and small molecules is an avenue which has enormous potential. To date there are >200 FDA approved proteins and peptides to treat a variety of diseases, but efficacy is often compromised by poor penetration across biological membranes, their immunogenicity and short plasma half-life (due to inadequate stability and rapid clearance) [16]. Therefore, the entrapment of proteins and peptides within nanocarriers has potential to overcome these limitations. This can be achieved by either encapsulating the protein/peptide within the nanoparticle itself or via chemical conjugation to the particle surface [17]. Moreover, studies have shown that hydrophobic ion pairing is a method in which the solubility of charged hydrophilic molecules can be modulated, where pairing with oppositely charged counter molecules (e.g. nuclear localisation sequence) can be utilised to increase the loading of peptides and nucleic acids within nanoparticle cores [18,19].

The use of nanoparticles and other nanomedicines as drug delivery systems is an approach now established clinically since the first FDA approval of a nanomedicine, DOXIL® (liposomal doxorubicin) in 1995 [20]. Since then, the field of nanomedicine has developed substantially, with several other nanomedicines entering the market. However, the application for use in non-cancer related conditions is also of particular interest, especially for vaccine delivery, notably implemented within the formulation of the numerous SARS-CoV-2 vaccines approved in 2021 [21–24]. More recently, the emergence of targeted therapies has significantly altered the design process of novel drug modalities, particularly when considering the treatment of solid tumours. These next generation nanoparticles are more frequently multifunctional, where their surface can be functionalised while also entrapping therapeutics within the nanoparticle, leading to more innovative approaches in actively targeting tumour sites and eliciting biological effects that have therapeutic usefulness, independent of enhanced delivery alone.

## Nanoparticles in cancer therapy

Despite the growth of the nanomedicine field and promising pre-clinical data, to date only 12 nanoformulations have been FDA/EMA approved for the treatment of cancer (Table 1).

**Table 1:** Advantages of clinically approved nanoparticles in the field of oncology

Name	Nanoparticle subtype	Clinical advantage	Cancer indication	Clinical approval
Doxil®	Pegylated liposomal (Doxorubicin)	Reduced systemic exposure	Ovarian cancer and multiple myeloma	FDA (1995) EMA (1996)
DanuoXome®	Non-pegylated Liposome (Daunorubicin)	EPR effect	HIV-associated Kaposi's Sarcoma various leukaemia's	FDA (1996)
DepoCyt®	Liposomal Cytarabine	EPR effect	Lymphoma Leukaemia	FDA (1999)
Myocet®	Non-pegylated Liposome (Doxorubicin)	Sustained release	Metastatic breast cancer	EMA (2000)
Abraxane®	Albumin bound nanoparticle (paclitaxel)	Sustained release	Metastatic breast cancer Metastatic pancreatic cancer Non-small cell lung cancer	FDA (2005) EMA (2008)
MEPACT®	Non-pegylated liposome (mifamurtide)	Sustained release	Osteosarcoma	EMA (2009)
NanoTherm®	Iron Oxide	Hyperthermic treatment	Brain tumours	EMA (2011)
Marqibo®	Non-pegylated liposome (vincristine sulfate)	Sustained release	Acute lymphoblastic leukaemia	FDA (2012)
Onivyde®	Pegylated Liposome (Irinotecan)	Immune evasion	Advanced pancreatic cancer	FDA (2015)
Vyxeos®	Liposome (cytarabine and daunorubicin)	Combination therapy Sustained release	Acute myeloid leukaemia	FDA (2017) EMA (2018)
Apealea®	Paclitaxel Micelle	EPR effect Sustained release	Ovarian, peritoneal, and fallopian tube cancer	EMA (2018)
NBTXR3®	Hafnium oxide NPs	Radiotherapy	Locally advanced soft tissue sarcoma	EMA (2019)

(This table summarised information which is available at accessdata.fda.gov (https://www.accessdata.fda.gov) and ema.europa.eu.en/medicines (https://www.ema.europa.eu/en/medicines) 22th September 2021).

Whilst initial successes in the clinic have predominantly used lipid-based carriers such as liposomes, many other opportunities exist through a broad range of polymers and materials which are FDA/EMA ‘*generally regarded as safe’* (GRAS) [25]*.* A growing area of interest focuses on the synthesis of polymeric nanoparticles as delivery systems where GRAS biodegradable synthetic polymers are commonly utilised (e.g. poly(DL-lactic acid) (PLA), poly(glycolic acid)(PGA) and poly(lactic-co-glycolic acid)(PLGA)) [26].

## Mechanisms of cellular targeting

### Passive targeting nanoparticles

**Figure 1.**
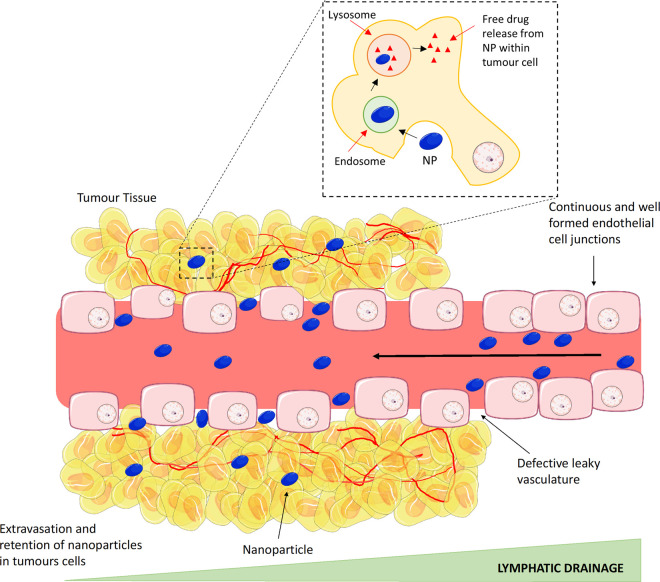
The enhanced permeability and retention (EPR) effect. During the initial stages of tumour formation, there is a rapid pro-angiogenic neovascularisation to supply the tumour with all nutritional requirements to survive. As a result of the rapid pace of formation, blood vessels are often defective, with large apertures left between endothelial cells resulting in a leaky vasculature. Nanoparticles have the capacity to exploit this defect in vessels, allowing them to pass from the blood vessels into the tumour site. Additionally, retention of the nanoparticles occurs within the tumour site as a result of poor lymphatic drainage. Image created with the use of Smart Servier Medical Art.

It is now well established that the presence of a leaky vasculature is a feature frequently associated within the hypoxic TME, where angiogenesis must occur simultaneously to fulfil the metabolic requirements of the rapidly proliferating tumour cells, resulting in a disorganised dense neovasculature. A particular feature of the majority of tumour vessels formed is that they are defective, with apertures (<600 nm) left between the endothelial cells [27]. Consequently, this leaky vasculature, coupled with poor lymphatic drainage in the tumour tissue, permits large macromolecules (>40 kDa) and nano-sized drugs to passively diffuse and accumulate at the disease site. This passive targeting phenomenon was first described in 1986 by Matsumura and Maeda and became known as the enhanced permeability and retention (EPR) effect [28–30] (Figure 1). Despite the approval of some nanoparticles which rely on the EPR Effect (e.g. Doxil © and Caelyx ©), robust exploitation of passive targeting for nanoparticle delivery has proven challenging. The foremost issue is the deep reliance of the EPR effect on intrinsic tumour biology, in particular: (1) intratumoural pressure, (2) degree of angiogenesis (endothelial gap size and blood supply), (3) stromal density of the tumour and perivascular growth [31]. As a result, the EPR effect is inevitably heterogenous with vast variation across tumours and individual patients [32–34]. Often only a small percentage (<1%) of nanoparticles accumulate within the tumour site [35], most often attributed to the multiple challenges aforementioned. Approaches to improve the EPR effect is an area of ongoing research with the use of vasodilating agents and vascular disruption to enhance uptake under investigation [36–40].

### Actively targeted nanoparticles

Since Paul Ehrlich suggested the concept of the ‘magic bullet’ in the early 1900's, where a therapeutic has the ability to selectively target diseased cells while avoiding/limiting intervention with healthy tissues, the concept of actively targeting tumour cells has been a focus of intense research [41]. Whilst nano-sized systems possess inherent delivery benefits, advances in synthesis/formulation approaches have resulted in the concept of active or site-specific targeting. This model has many benefits and is particularly relevant in the treatment of cancer, where the ability to selectively target the tumour site is desirable to reduce off-target toxicity towards healthy tissues.

**Figure 2.**
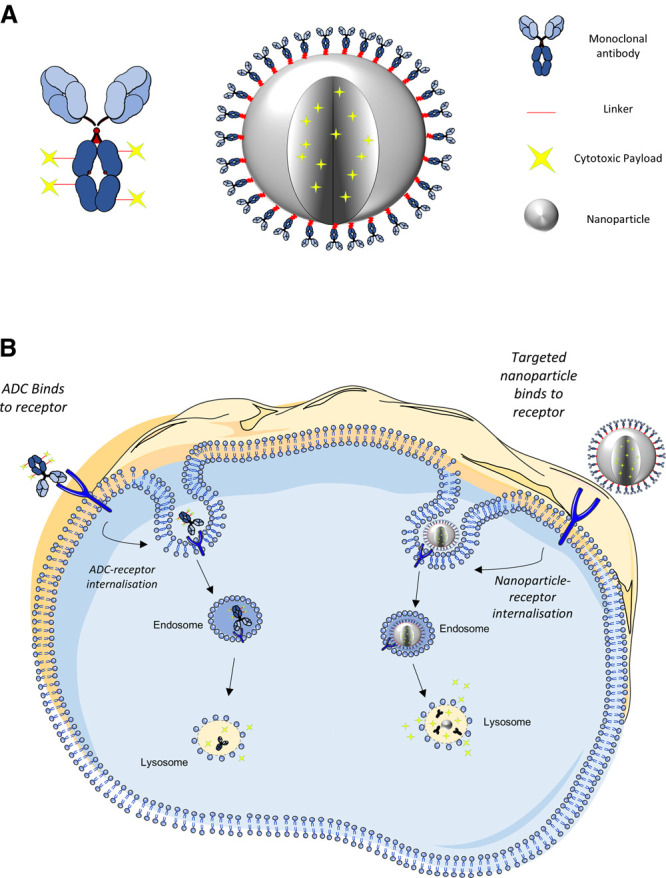
Structure, binding and internalisation of Antibody-drug conjugate's (ADCs) and multifunctional antibody conjugated nanoparticles. Schematic representation highlighting (**A**) the conceptual structure of an ADC and a multifunctional antibody conjugated nanoparticle and (**B**) internalisation, breakdown, and drug release within the cell. The tumour-specific ligand of the ADC or nanoparticle interacts with and binds to the cell surface expressed antigen. Upon binding, the antigen–protein complex becomes internalised via receptor mediated endocytosis. The nanoparticle or ADC linker is degraded within the endo-lysosomal system, resulting in drug release within the cell. Illustrations not to scale. Image created with the use of Smart Servier Medical Art.

Active targeting has already been used with success in the clinic, with a total of 11 antibody-drug conjugates (ADCs) approved (e.g. Adcetris™ and Kadcyla™) [42,43]. ADCs use monoclonal antibodies linked to a cytotoxic payload, where the antibody is employed to specifically target and deliver the payload to be internalised and released within the target cell of interest (Figure 2A). ADCs are becoming an important therapeutic development approach, proving a favourable treatment option for a variety of cancer patients. Indeed, one specific attribute of these drugs is that they can overcome resistance to a cell target, providing a second line treatment to patients who have acquired resistance. The classic example of this is the treatment of Herceptin^TM^ resistant Her2 positive breast tumours with Kadcyla^TM^, which uses Herceptin as the targeting agent to deliver the microtubule inhibitor maytansine payload [16]. ADC development includes the development of stable linkers adopting more innovative site-specific conjugation methods to improve conjugation stability, as well as reducing variability and optimisation of Drug:Antibody ratios (DAR). The conjugation characteristics of the linker are vital in controlling the therapeutic window and effectiveness of the ADC, where the DAR determines overall potency and toxicity. DAR represents the average number of cytotoxic drug molecules attached per single antibody (typically <10) which means that the key limiting factor for ADC effectiveness is receptor copy number on the target cell; therefore, the lower the receptor density on the cell surface, the less drug will be successfully internalised. Many studies have shown that increasing the DAR can destabilise the antibody and therefore ADCs normally explore the use of high-potency payloads to compensate, which inherently have narrower therapeutic windows [44]. The majority of ADCs under investigation utilise potent microtubule inhibitors, yet many have failed to reach the clinic due to their dose limiting toxicities. In an attempt to overcome this, current research focuses on the use of less potent cytotoxic drugs, with a lower IC_50_ than maytansine or auristatin, such as topoisomerase targeting agents (e.g. camptothecin derivatives), highlighted by the recent FDA approval of trastuzumab deruxtecan (approved 2019), and more recently, Sacituzumab Govitecan, (approved 2021) an ADC which targets Trop2 linked to SN-38, the active metabolite of irinotecan [45–48].

Following on from the experience of developing ADCs, the ability to conjugate targeting moieties such as antibodies to the surface of nanoparticles represents an exciting approach to develop a new type of ‘magic bullet' therapy [49] (Figure 2A). As with ADCs, the binding of the tumour-specific antibody with its cognate antigen on the diseased cell can trigger internalisation of the complex to deliver the payload internally and elicit its cytotoxic effect, thus limiting payload exposure to healthy tissues [50] (Figure 2B). An interesting facet of antibody targeted nanoparticles is that they have the potential to enhance the DAR which remains one of the key efficacy-limiting factors with ADCs. This is because nanoparticles can be packed with an increased payload resulting in DARs of >100 as opposed to <10 with current ADC technologies, ensuring the internalisation of a much higher concentration of drug, which compensates for limitations such as low copy number [49].

Despite the clear opportunities provided by the exquisite selectivity and affinity of antibodies, it is important to realise that they are only one class of agent that can be used to target nanomedicines. In the following sections, the application of antibodies and other targeting agents will be further discussed.

## Functionalisation of nanomedicines

**Figure 3.**
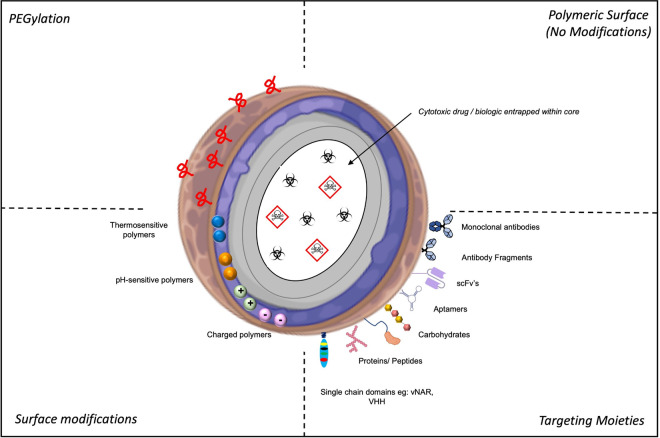
Different strategic approaches for the surface functionalisation of nanoparticles. Nanoparticle surfaces can be modified through the incorporation of different polymers/lipids to modify surface charge as well as thermo and pH sensitive properties. PEGylation of the nanoparticle surface can be used to extend half-life of the nanoparticles *in vivo*. Furthermore, nanoparticles can be targeted through the conjugation of targeting moieties to the nanoparticle surface. In addition to surface modification, nanoparticle design can vary based on the nature of the therapeutic agent entrapped within the nanoparticle core. Abbreviations: PEG, Polyethylene glycol; vNAR, Variable new antigen receptor; scFv, single-chain variable fragment; sdAb, single domain antibodies. Image created with the use of Biorender & Smart Servier Medical Art.

The surface of a nanoparticle represents a bioengineer's dream! The ability to coordinate the presentation of functional handles on the nanoparticle surface that can be modified in a range of different ways to achieve the exact specification required for their intended use is potentially unlimited. Not only can nanoparticles be functionalised for use in active targeting; nanoparticle surfaces can be further manipulated to change surface charge and incorporate stimuli responsive properties (Figure 3).

### PEGylation

For polymeric nanoparticles to reach their target tissue they should be capable of avoiding clearance through the mononuclear phagocytic system (MPS). The MPS functions to remove foreign material from the blood stream and is the first barrier which nanoparticles encounter *in vivo*. Through opsonisation, immunoglobulins and complement system proteins adhere to the hydrophobic nanoparticle surface effectively ‘flagging’ them for recognition by macrophages and removal via phagocytosis [51]. To avoid/limit MPS uptake, researchers have developed a strategy which involves coating the surface of the nanocarrier with a hydrophilic polymer. The presence of hydrophilic PEG chains on the hydrophobic nanoparticle surface creates a barrier which permits the nanoparticle to evade opsonisation through camouflaging the surface charge and steric repulsion, limiting the ability of opsins to bind to the nanoparticle surface [52]. This significantly reduces the rate at which nanoparticles are removed from the blood stream [53]. Whilst a range of polymers may be utilised, PEG is by far the most commonly used as it is generally regarded as safe in formulations with an extensive track record in a variety of clinically approved drugs [5,52].

Many studies have illustrated the integration of PEG within formulations to increase circulation time for both liposomes and polymeric nanoparticles [54,55]. Importantly, the use of PEGylated products is now well established in the clinic. Notably, the first formulation to be approved by the FDA, Caelyx® (DOXIL, 1995), is a doxorubicin entrapped PEGylated liposome. *In vivo* studies demonstrated clearly that the serum half-life of the doxorubicin payload could be significantly enhanced by the addition of PEGylation in the formulation [20,56]. Additionally, PEGylated liposome formulations also have an increased ability to penetrate the leaky tumour vasculature, thus resulting in enhanced drug accumulation [56–58]. More recently, PEGylated formulations such as Onivyde® (PEGylated Irinotecan liposome) for the treatment of advanced pancreatic cancer have been approved, whilst Genexol® (PEG-PLA micelles containing paclitaxel) for the treatment of metastatic breast cancer is currently under clinical investigation [15,53,59–61].

### Targeting moieties

This approach requires the presence of a target antigen which is highly or uniquely expressed on tumour cells in comparison with normal healthy tissues, as well as the identification of a targeting moiety which binds with high affinity and specificity to the target antigen selected [50]. The ideal targeting ligand should possess a functional handle which aids stable conjugation without diminution of ligand affinity. Conjugation should result in targeted nanoparticles which display minimal aggregation and have an adequately dense coating of the targeting moieties [62]. To date, a wide range of targeting moieties have been studied to functionalise nanoparticles including traditional antibodies formats, F(Ab)_2_ fragments, proteins, peptides and aptamers to more innovative approaches through the use of small single chain antibodies such as ScFv's, VHH domains and vNARs [63–69]. When exploiting such ligands for the targeted delivery of nanoparticles, it is often necessary to introduce the appropriate reactive moiety on the surface of the nanoparticle, to facilitate conjugation. Countless bioconjugation methods may be applied, with the most common approach being the use of carbodiimide and maleimide chemistries. However, alternative approaches, including ‘click' chemistries, are now being evaluated with superior conjugation efficiencies and biological functionalities reported [64,65,70–72].

**Figure 4.**
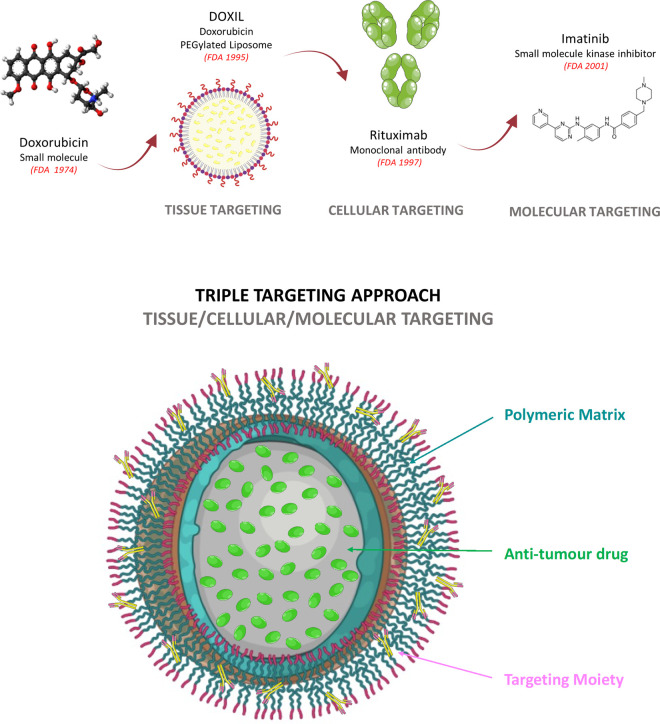
Evolution of targeted therapies from the approval of small molecules to the first approval of nanomedicines and molecular targeting small molecule kinase inhibitors. A schematic representation of the progression of targeted therapies from the use of small molecules alone, through to targeting at a tissue, cellular and molecular level. Such advancement has paved the way for potential triple targeting approaches, wherein future targeted nanoparticle formulations, possessing a range of targeting moieties and advanced polymer chemistries, may target all three levels simultaneously and in turn greatly enhance treatment specificity and efficacy. Image created with the use of Biorender & Smart Servier Medical Art.

A significant volume of research has been carried out investigating surface functionalisation of nanoparticles. BIND-014 (Accurins), a polymeric nanoparticle with a therapeutic payload (Docetaxel) designed to target tumours with an anti-PMSA (Prostate-specific membrane antigen) targeting ligand grafted on the nanoparticle surface was the first to enter clinical trials [73,74]. Despite not achieving clinical approval, experience gained from the development of BIND-014 has highlighted the need for a programmable nanoformulation, a combination of controlled release polymer and a targeting moiety which has the capacity to target the tumour at three levels: (1) tissue, (2) cellular and (3) molecular (Figure 4).

As a result, many researchers have exploited this model of triple targeting, where studies have indicated that conjugation of Trastuzumab (anti-HER2) or Rituximab (anti-CD20) to PLA-nanoparticles results in a six-fold increase in the rate of nanoparticle uptake compared with similar controls lacking antibody functionalisation [75]. Further studies have focused on optimising the conjugation of both Trastuzumab and Cetuximab F(ab)_2_ fragments to PLGA-PEG nanoparticles, through a selectively re-bridged strained alkyne handle on the F(ab)_2_ fragments to permit azide ‘click’ conjugation chemistry [65,76]. This advanced conjugation method resulted in significant improvement in antigen binding owing to the production of a highly uniform nanoparticle where the targeting moiety is conjugated in a site-selective and optimally orientated manner, when compared with conventional amine-selective carbodiimide chemistry [71]. In addition to the use of monoclonal antibodies and fragments, research has also more recently focused on the use of antibody like molecules (e.g. vNARs), peptides and aptamers as next generation targeting agents [77,78]. As a result of their unique properties these new targeting agents have the capability to enhance tissue penetration and extend tumour retention times, improving upon previously utilised nanoparticle targeting ligands [79,80]. Whilst their potential use for nanoparticle functionalisation has already been demonstrated within the literature, they are yet to enter clinical investigation [63,64,81–83].

The role of active targeting can be further enhanced in some settings through receptor activation, which can be utilised as a dual approach concurrently with active targeting. Some receptor targeted monoclonal antibodies have showed limited efficacy in clinic (e.g. AMG655 and Death Receptor 5 (DR5)), which is thought to be as a result of weak receptor activation [84]. For instance, DR5 requires cross-linking or receptor clustering for activation and therefore recent DR5-targeted therapeutic design has progressed towards multivalent agents, whereas first-generation DR5-targeting antibodies were bivalent [85–87]. Alternatively however, through the use of a multi-functional antibody-conjugated nanoparticle, a high number of antibody molecules can be conjugated per particle, resulting in a sufficient density of antibody paratopes to induce apoptosis via effective DR5 clustering, unlike free antibody alone [66,88,89]. Furthermore, studies have indicated that the entrapment of a cytotoxic payload can enhance drug targeting to antigen-expressing cells resulting in superior toxicity [65].

### Surface charge

**Figure 5.**
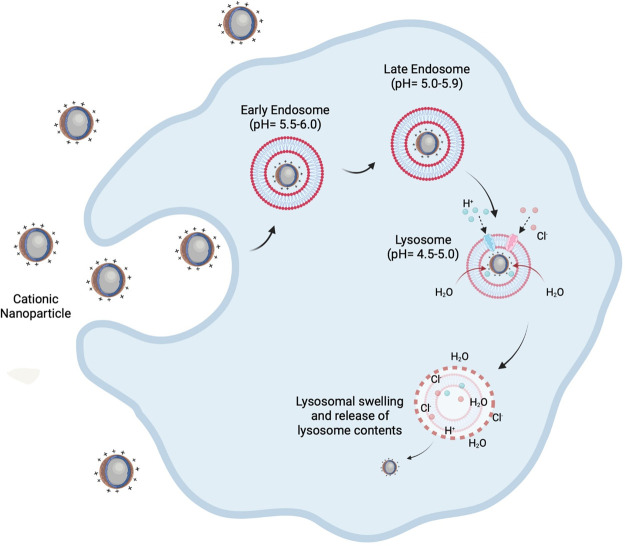
The proton sponge theory. Cationic nanoparticles have the ability to escape the endo-lysosomal pathway, allowing cargo to enter the cytosol using methods such as the proton sponge effect. Endosomes fuse into lysosomes, where unprotonated amine groups on cationic nanoparticles become protonated. The protonation results in the influx of chlorine ions and water causing the osmotic rupturing of the lysosomal membrane, stimulating stimulate cargo release. Image created with the use of Biorender.

Altering the surface charge of nanoparticles is another potential avenue to tailor nanoparticle delivery systems, where nanoparticles can possess an anionic, cationic, or neutral charge. To date, the use of anionic/neutral nanoparticles have shown success within the clinic (Table 1), however utilisation of cationic nanoparticles is of much interest due to their ability to enhance cellular uptake (in comparison with neutral/anionic nanoparticles), and manipulate intracellular trafficking once internalised (e.g. endo/lysosomal escape) [90]. Cationic nanoparticle interactions with the cell are promoted via electrostatic attraction owing to the net negative charge which is characteristic of the plasma membrane. This in turn enhances clathrin-mediated endocytosis of the nanoparticle, when compared with anionic or neutral nanoparticles [91]. Additionally, studies have indicated uptake may also be ATP-independent as a result of direct translocation into the cell [90,92–94]. One drawback however in the use of cationic nanoparticles is the associated increased cell cytotoxicity, which is significantly increased compared with anionic/neutral nanoparticles. The exact mechanism of action associated with the toxicity is not fully elucidated, however it has been suggested that cationic nanoparticles activate caspase 3/7 and cleave PARP, signifying stimulation of apoptosis and cell necrosis. In addition to cytotoxicity, cationic nanoparticles are more rapidly cleared from the blood stream as a result of their charge, however this can be lessened by incorporation of PEG within the nanoparticle formulation to increase plasma circulation time, as discussed above. Aside from cationic nanoparticle toxicity and clearance, with the correct formulation, cationic nanoparticles may reduce cargo degradation and elicit desired intracellular effects at a rapid pace via escaping the endo-lysosomal pathway. This can be achieved through exploiting phenomenon such as the proton sponge effect, where pH buffering agents can be manipulated due to the acidic nature of endosomal/lysosomal vesicles to stimulate cargo release as a result of membrane destabilisation [95,96]. Under acidic conditions, numerous macromolecules which exhibit low pKa amine groups (e.g. PEI) promote release, whereby unprotonated amines within the cationic polymer become protonated, which in turn leads to an influx of chlorine ions and water into the lysosome. This influx of water results in osmotic swelling and lysis, whilst paired forces of repulsion in the cationic polymer results in further disruption of the lysosomal membrane (Figure 5) [96–99].

### pH/thermo sensitive nanoparticles

Stimuli responsive nanoparticles have generated interest due to their potential to improve drug delivery by releasing therapeutics in response to pH and/or temperature alterations. pH sensitive nanoparticles are often tailored to respond to a fluctuation in pH found within the TME, in particular the acidified vesicles of the endo/lysosomal systems where nanoparticles accumulate following endocytosis. Thus pH-sensitive polymers such as PDPA (poly(2-diisopropylamino)ethyl methacrylate) and PPAA (Poly(propylacrylic acid) can be utilised to exploit the acidic pH of endosomes and lysosomes [99–101]. Cytosolic delivery of therapeutic agents from the endosome is crucial in order to protect drugs susceptible to degradation by lysosomal enzymes. In such cases, nanoparticle formulation can be tailored through incorporation of polymers which disassemble at endosomal pH, promoting endosomal escape, or via the incorporation of cell penetrating molecules, such as peptides which exclusively promote endosomal escape [102]. Activated upon a fall in pH (4.5–6.0), these nanoparticles enable the release of their cargo within these more acidic environments and subsequent release into the cytoplasm [103,104]. Furthermore, thermosensitive liposomes (e.g. ThermoDox), modified via the addition of thermosensitive lipids (e.g. distearoyl phosphocholine and 1,2-dipalmitoyl-sn-glyce-ro-3-phosphocholine) and/or polymers [e.g. poly(N-isopropylacrylamide)] are considered favourable for site-specific delivery, where particles remain in a stable state at their lower critical solution temperature. Upon undergoing slight change in temperature, most often induced by radiofrequency ablation, which induces mild hyperthermia of the tumour site (∼40–43°C), thermosensitive nanoparticles have the ability to trigger cargo release predominantly within the TME [105,106]. However, issues have been raised on delivery effectiveness, as localised therapy depends on the ability to accurately maintain a controlled elevated temperature, specifically within the tumour site while minimising exposure to surrounding healthy tissue. As a result, the ability to target deep within tissues has proven problematic, thus currently limiting treatment to superficial tumours. However research is progressing through the use of focused high density ultrasound to allow more controlled and specific targeting [107].

## The future of nanomedicine

The amalgamation of a small size, high surface area: volume ratio and ability for extensive functionalisation endow nanoparticles with the capability to address a range of drug delivery challenges [1,8]. However, successful clinical application of nanomedicines has been limited. Whilst the total number of nanoformulations which are FDA approved has increased in the last decade, many of these are for non-biomedical implications. Whilst early-stage research demonstrates the advantages of nanomedicine delivery approaches, several issues must be addressed to translate this more readily from bench to clinic, such as better understanding the biological fate of nanomaterials *in vivo,* better appreciation of their interactions within the blood stream and further investigation of their trafficking through intracellular compartments. In addition to investigations into their biological fate, issues relating to manufacturing on large scale must also be considered. Whilst complexity of design may theoretically aid delivery, this must be implemented in a manner which is both reproducible and scalable. Such manufacturing issues have recently been addressed through the advancement of microfluidic technology, which holds great promise for nanomedicine production at an industrial scale [108,109]. Whilst nanomedicines have been granted approval in the oncology setting, advancements of the field such as improved targeting strategies, has led to the development of innovative formulations, some of which are now undergoing clinical trials (Table 2).

**Table 2:** A promising future: a selection of nanoparticles currently undergoing clinical trials within the field of oncology (with clinical stage)

Name	Nanoparticle subtype	Clinical advantage	Cancer indication	Status	Clinicaltrial. gov identifier
MM-310	Liposome (Ephrin type-A receptor targeted docetaxel)	Targeted therapy	Solid tumours	Phase I ongoing	NCT03076372
PROMITIL	Pegylated liposome (mitomycin-C)	Immune evasion	Solid tumours	Phase I complete Phase Ib recruiting	NCT03823989
ANTI-EGFR-IL dox	Liposome (Doxorubicin) Anti-EGFR	Targeted therapy	Advanced triple negative EGFR positive breast cancer	Phase I/II recruiting	NCT02833766 NCT03603379
ThermoDox	Liposome Thermosensitive (Doxorubicin)	Temperature triggered sustained release	Breast Cancer Liver tumours Hepatocellular carcinoma	Phase I/II/III	NCT02536183 NCT02181075 NCT02112656 NCT00826085
Genexol-PM	Polymeric micelle nanoparticle (Paclitaxel)	Sustained release	Ovarian cancer	Phase II	NCT01276548
NK105	Polymeric nanoparticle (Paclitaxel)	Sustained release	Breast cancer	Phase III	NCT01644890
CTX-Somatostatin	Polymeric nanoparticle (Cetuximab), somatostatin decorated	Targeted therapy Sustained release	Colon cancer	Phase I	NCT03774680
PRECIOUS-01	PLGA nanoparticle (immunomodulating agent)	Sustained release Cargo delivery	NY-ESO-1 positive cancers	Phase I	NCT04751786
AR160	Paclitaxel albumin-stabilized/Rituximab-coated nanoparticle	Targeted therapy Sustained release	Non-Hodgkin's lymphoma	Phase I	NCT03003546
NK012	Polymeric nanoparticle (SN38)	Sustained release	Solid tumours	Phase II	NCT00951613 NCT00951054
Anti-EGFR-IL-dox	EGFR-targeting liposome (Doxorubicin)	Targeted therapy Sustained release	Solid tumours Breast cancer	Phase I Phase II	NCT01702129 NCT02833766

(This table summarised information which is available at Clinicaltrials.gov (https://clinicaltrials.gov) as of 22nd September 2021).

Advanced nanotechnology provides the opportunity to combine numerous treatment avenues within the same nanoparticle to develop multifunctional nanoparticles combining chemotherapies, targeting agents, immunotherapies and photodynamic therapies, highlighting the potential scope for ‘precision' nanoparticle development, which could be particularly important for addressing the need of targeting metastatic cancer in complex evolving tissue milieu [110]. Furthermore, nanoparticles can be designed to respond to specific stimuli, such as fluctuations in temperature or pH, as well as hypoxia and the presence of specific enzymes within the TME [110–115].

In addition to chemotherapeutic indications; immune therapy, gene delivery and gene silencing are rapidly emerging treatments within the field of oncology, offering a variety of advantages including high specificity, potency, reduced off-target effects, as well as the possibility of targeting multiple genes, thereby concurrently targeting many of the hallmarks of cancer [116,117]. Immunotherapy is a catch-all term that involves harnessing or manipulating the patient's own immune system to not only to recognise but kill cancer cells. Nanomedicine may offer opportunities to enhance this revolutionary approach, for example through delivering chemotherapies such as oxaliplatin which can induce immunogenic cell death, thus not only killing the tumour cell, but changing the TME to unleash an anti-tumour immune response [118]. Additionally, surface modification of nanoparticles to incorporate immune stimulation such as the use of an anti-CD40 monoclonal antibody, has been shown to induce potent CD8+ T cell responses resulting in tumour growth attenuation and prolonged survival in preclinical models [118,119].

Other than the more conventional use for entrapment of APIs, nanocarriers have the potential to target specific genes. Gene therapy has shown great promise in the literature, both as single agents and in combination with cytotoxic agents, where RNA molecules such as small interfering RNAs (siRNAs) and micro-RNAs (miRNAs) are capable of regulating expression of certain genes which contribute to cancer development. Despite preliminary success, clinical application remains largely limited; a result of poor pharmacokinetics and potency as well as off target effects and intolerable levels of toxicity. Delivery of large negatively charged nucleic acids which are fragile inside the cell can also be problematic, therefore the use of a nanocarrier could be a solution to protect the cargo while aiding delivery to the TME as well as minimising toxicity [120]. Since the introduction of the first liposome based siRNA delivery system in clinical trials in 2008, the development of DNA/RNA based nanocarriers has been an evolving field [121]. Studies have looked at systemic delivery of RNAs using scFv modified liposome-polycation-hyaluronan nanoparticles in lung cancer models, which through inducing apoptosis led to significant tumour inhibition [122]. Additional studies have investigated the use of a nanosized immunoliposome-based anti-HER-2 siRNA delivery complex to preferentially target tumour cells, where efficiency of the complex was improved by the inclusion of a pH-sensitive histidine-lysine peptide [123]. The complex demonstrates the ability to sensitise tumours to chemotherapeutic agents and silence the target gene of interest, effecting downstream pathways *in vivo*, as well as significantly inhibiting tumour growth in a pancreatic cancer model [123].

Within the past year, the subject of mRNA has become more of a ubiquitous topic, owing to the approval of RNA-based vaccine nanocarriers for COVID-19 worldwide [23]. The remarkable speed at which these vaccines were advanced is majorly owed to the fact that delivery of nucleic acids using lipid nanoparticles has been widely examined, where structure, uptake, surface-functionalisation endosomal escape, cargo release, clearance and, importantly, safety had to a degree already been optimised. This recent interest in nanomedicines highlights the abundance of research which is on-going, where combination with other therapies as well as further optimisation of the delivery system may hold the key to improve mRNA-based therapies and gene editing.

## Conclusion

Despite many advances in cancer treatment, there is still a clinical need for superior, kinder, and more effective therapies. The extensive application of nanomedicines within oncology has yielded some significant benefits but there is much greater potential to be still realised. With formulation advancements in terms of entrapping therapeutics within the nanoparticle core, as well as the concept of surface functionalisation aiding active targeting, an ever-growing number of formulations are likely to enter clinical trials for the treatment of cancer in the coming years. Further work to better understanding the biological fate of nanomaterials *in vivo* as well as improvements on large scale manufacturing and stability will further increase the success of the nanomedicine field within oncology.

## Perspectives

- In summary, nanoparticle drug delivery systems offer significant advantages when compared with their free drug counterparts, overcoming limitations in delivery via controlling the biological fate of drugs *in vivo*, including higher drug deposition at the tumour site, in turn increasing drug efficacy and reducing undesirable off-target side effects.
- Advancement within the field, particularly in regard to improved targeting strategies, has led to the development of innovative formulations, with a particular focus on surface functionalisation using targeting ligands, many which are currently in clinical trials.
- Whilst 12 nanomedicine therapies have been clinically approved for an oncology indication, these have primarily focused on entrapment and PEGylation to improve API performance. Therefore, combination of entrapment with advancements in active targeting and surface modifications of nanomedicines have the potential to realise tangible patient benefit via exploiting a triple targeting approach. This, in combination with improved manufacture scale-up to ensure a commercially viable system, should facilitate the advent of next generation nanomedicines into the clinic.

## Open Access Statement

Open access for this article was enabled by the participation of Queen's University Belfast in an all-inclusive Read & Publish pilot with Portland Press and the Biochemical Society under a transformative agreement with JISC.

## Abbreviations

ADCs: antibody-drug conjugates
API: active pharmaceutical ingredient
DAR: Drug:Antibody ratios
DR5: Death Receptor 5
EPR: enhanced permeability and retention
MPS: mononuclear phagocytic system
PGA: poly(glycolic acid)
PLA: poly(DL-lactic acid)
PLGA: poly(lactic-co-glycolic acid)
TME: tumour microenvironment
